# Zr-Site Lewis Acidity Determines Terpenoid Reduction Selectivity

**DOI:** 10.1021/acscatal.5c07220

**Published:** 2026-02-05

**Authors:** Kinga Gołabek, Svetlana Kurucová, Juan Francisco Miñambres, Klára Veselá, Talat Zakeri, Jan Přech

**Affiliations:** Department of Physical and Macromolecular Chemistry, Faculty of Science, 112302 Charles University, Albertov 6, Prague, Praha 128 43, Czech Republic

**Keywords:** transfer hydrogenation, MPV reduction, lewis acidity, Zr-zeolite, citronellal, citronellol, isopulegol, acetone adsorption

## Abstract

Lewis acid zeolites, primarily Al-free Zr and Sn silicates, catalyze the chemoselective reduction of ketones and aldehydes to the corresponding alcohols through hydrogen transfer (Meerwein–Ponndorf–Verley (MPV) reduction). Sn silicates are more active in the MPV reduction of ketones, whereas Zr silicates are more active in the MPV reduction of aldehydes. However, the catalytic activity of these zeolites has not been accurately ascribed to “open” vs. “closed” Zr sites even though this correlation is crucial for systems whose substrate structure allows competing reaction pathways. For example, MPV reduction of citronellal competes with carbonyl-ene cyclization to isopulegol and acetalization in the citronellal reaction with 2-propanol. Therefore, we aimed to correlate thoroughly characterized Lewis acid sites in Zr-substituted beta and MFI zeolites with their selectivity. For this purpose, we analyzed Zr-zeolite acidity by fourier transform infrared spectroscopy (FTIR) spectroscopy of adsorbed deuterated acetonitrile and acetone because deuterated acetonitrile probes “open” Zr sites without interacting with “closed” sites, but acetone identifies both “open” and “closed” sites. Our results showed that Zr-beta rich in Zr “closed” sites favored MPV reduction. Conversely, Zr-beta rich in “open” sites and reference catalysts yielded isopulegol as the main product. Ion exchange of the Zr-beta “open” sites with Na^+^ cations deactivated these sites, thereby switching the selectivity to citronellol. In turn, the silanol groups of the catalyst promoted acetalization, regardless of substituting the heteroelement (Zr or Sn). These findings demonstrate that Zr-site Lewis acidity determines terpenoid reduction selectivity, as the relatively weaker Zr-beta “closed” sites catalyze citronellal MPV reduction to citronellol, while the relatively stronger Zr-beta “open” sites catalyze intramolecular carbonyl-ene cyclization to isopulegol. Moreover, this correlation between selectivity and Zr-site Lewis acidity may enable us to design specific catalysts, even for systems with competing reactions, based on quantitative data acquired using our experimental paradigm.

## Graphical abstract

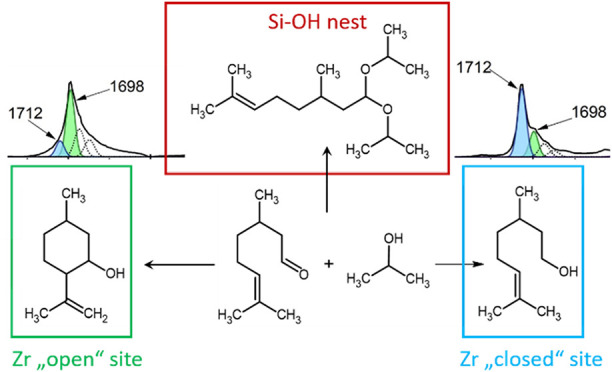

## Introduction

Zeolites substituted with tetravalent metal ions (e.g., Ti^4+^, Sn^4+^, Zr^4+^, and Hf^4+^) have Lewis acid properties,^1^ catalyzing a wide range of chemical transformations, such as hydrogen transfer reactions (Meerwein–Ponndorf–Verley reaction^2^ and sugar isomerization^3^), epoxidation with peroxides (titanosilicates only),^4^ Baeyer–Villiger oxidation (tin silicates only),^5,6^ and dehydration reactions.^7^ Among these reactions, Meerwein–Ponndorf–Verley (MPV) reduction stands out for enabling the chemoselective transformation of aldehydes and ketones to the corresponding alcohols, avoiding C=C saturation, under mild reaction conditions, that is, at low temperatures (<100 °C) and without any pressure apparatus or molecular hydrogen.^8,9^ Natural compounds, such as terpenoids, can be unstable or undergo undesirable reactions at high temperatures. Accordingly, these compounds may be efficiently transformed by MPV reduction.

MPV reduction is a hydrogen transfer reaction of a carbonyl compound and a sacrificial alcohol that serves as a hydrogen donor. Weak Lewis acidic metal oxides, such as ZrO_2_, catalyze this reaction, but only at high temperatures (>100 °C),^10,11^ so the MPV reduction of ketones is typically performed over Sn zeolites, mainly over Sn-beta, which is more efficient than Zr-beta and Ti-beta.^12,13^ Conversely, in the MPV reduction of aldehydes, the order of reactivity is reversed (Hf-beta > Zr-beta > Sn-beta).^14^ MPV reduction is tolerant to many functional groups,^13^ and C=C double bonds are not saturated in this reaction, but its selectivity decreases when the substrate structure allows Lewis acid to catalyze competing reactions such as carbonyl-ene reaction.^15^ Such a reaction system is exemplified by citronellal MPV reduction, where the MPV reaction yields citronellol, while the competing carbonyl-ene intramolecular reaction results in a pool of isopulegol isomers. In this reaction system, selectivity varies with the catalyst,^16,17^ suggesting that the Lewis acidity of these zeolites determines their catalytic efficiency and selectivity.

The catalytic properties of Lewis acid zeolites are, in fact, governed by the strength, geometry, and confinement of their acid sites and by the hydrophilicity of their environment.^18,19^ Such properties are primarily affected by the incorporated metal, catalytic site connectivity, and, to some extent, zeolite structure. Each zeolite with an incorporated metal can have at least 3 types of acid sites: (i) a “closed” site where the metal ion is connected via 4 oxygen bridges to the neighboring SiO_4_ tetrahedra; (ii) an “open” site where the metal ion is connected via 3 oxygen bridges, with two −OH groups replacing the fourth oxygen bridge; and (iii) a “double-defective” site with only 2 oxygen bridges (Scheme 1).^1^ These 3 types of sites can be distinguished and, in theory, quantified by Fourier transform infrared spectroscopy (FTIR) analysis of adsorbed probe molecules. Deuterated acetonitrile (*d*_3_-acetonitrile) is used for Sn-substituted zeolites,^20^ while carbon monoxide^21^ or other carbonyl probes^22,23^ are used for Zr-zeolites because acetonitrile does not interact with Zr “closed” sites.^21^ Using these probes, studies have shown that the relative acid site strength decreases in the following order: “open” site > “closed” site > “double-defective” site. Sn “open” sites catalyze sugar isomerization reactions,^24^ which are, in principle, intramolecular MPV reductions, whereas Sn “closed” sites catalyze dehydration reactions.^25^ So, the catalytic activity of Sn zeolites may be intrinsically correlated with the prevailing type (“open” vs. “closed”) of their Lewis acid sites.

**Scheme 1:**
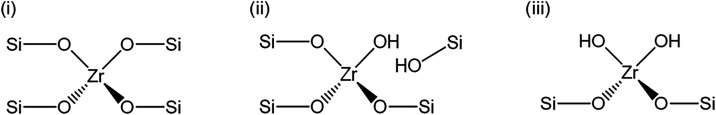
(i) “Closed”, (ii) “Open”, and (iii) Double-Defective Zr Lewis Acid Site

Active site accessibility is another defining parameter of the overall catalytic activity. The most commonly used Lewis acid zeolite is zeolite beta (IZA code: *BEA), a large-pore zeolite with a 3-dimensional system of intersecting 0.75 × 0.57 nm and 0.65 × 0.56 nm pores.^26,27^ But with bulky substrates, like many natural compounds, the reaction can be slowed down or limited to the external surface of zeolite crystals because reactants can only slowly penetrate the channel system or are unable to access the channel system at all. And even inside zeolite micropores, transition-state shape-selectivity can affect the catalytic activity. To compensate for poor acid site accessibility,^28^ 2-dimensional (layered) zeolites with an extremely short diffusion path and a high external surface area^29–31^ have been prepared by direct synthesis with isomorphously incorporated Sn atoms, namely, self-pillared pentasil MFI/MEL intergrowths.^32^ These zeolites catalyze reactions such as lactose-to-lactulose (disaccharide) isomerization.^32^ Conversely, restricting the channel size may limit some reaction pathways. For instance, the confined space in the zeolite beta channels favors the formation of *cis-*4-*tert*-butylcyclohexanol in the MPV reduction of 4-*tert*-butylcyclohexanone, although the *cis* isomer is less thermodynamically favorable than the *trans* isomer.^33,34^ Therefore, while it is primarily determined by the type and strength of the Lewis acid sites, catalytic selectivity can be further influenced by shape-selectivity effects.

In this contribution, we aim at separating the contribution of these two factors (type of Lewis acid site and channel size restriction) and correlating the type of Zr Lewis acid site with the selectivity of three competing reactions, citronellal MPV, carbonyl-ene reaction, and acetalization (Scheme 2). Zr silicates are known to be more active in the MPV reduction of aldehydes, but the catalytic activity of these zeolites has not been accurately ascribed to “open” vs. “closed” Zr sites despite the importance of this correlation for systems with competing reaction pathways. To identify this correlation, we performed MPV reactions over Zr-beta zeolites with similar Zr content but different relative concentrations of “open” and “closed” Lewis acid sites. These Lewis acid sites were analyzed by FTIR spectroscopy of adsorbed *d*_3_-acetonitrile and acetone. Both catalytic and spectroscopic results were compared with data on conventional and pillared Zr-MFI and Sn-beta zeolites, used as references. By ascribing each reaction pathway to a type of acid site, we not only enhance our understanding of zirconosilicate zeolite catalytic properties but also may facilitate the design of specific catalysts, even for systems with competing reactions based on quantitative data.

**Scheme 2:**
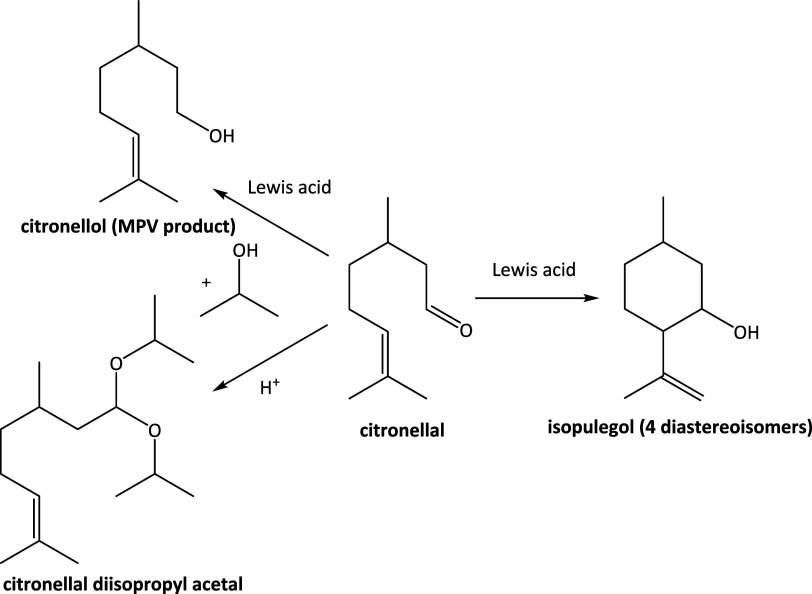
Reaction pathways of citronellal transformation in 2-propanol over Lewis acid zeolites.

## Experimental Section

### Preparation of the Catalysts

#### Beta Zeolites

Zr-beta-A, Zr-beta-B, and Zr-beta-C zeolites were prepared by seed-assisted hydrothermal synthesis. Zeolite beta seeds were hydrothermally synthesized by dissolving 0.019 g of aluminum powder (>93%, Penta, Czech Republic) in 14.73 g of aqueous 35% tetraethylammonium hydroxide (TEAOH, Sigma-Aldrich). This aluminate-containing solution was added into a mixture of 14.73 g of 35% TEAOH and 32.11 g of tetraethyl orthosilicate (TEOS, 100%, VWR Chemicals) and stirred for 18 h, subsequently adding 3.18 g of a 48% HF aqueous solution (Emsure). The final mixture was transferred into a Teflon-lined steel autoclave and kept at 140 °C in an oven with agitation (60 rpm) for 3 days.^35^ The resulting solid was filtered, washed with 500 mL of deionized water, dried (60 °C, overnight), and calcined in air at 580 °C (2 °C/min temperature ramp) for 6 h. The calcined zeolite beta was dealuminated by acid treatment with 1 M HNO_3_ (65%, P.A., Lachner) (30 g of solution per 1 g of zeolite) for 4 h at 60 °C to obtain beta seeds.^36^ These dealuminated beta seeds were filtered, washed with deionized water, and dried at 60 °C. ICP-MS elemental analysis of the seeds (see conditions below) showed that Si/Al > 500, thus confirming complete dealumination.

Zr-beta-A, Zr-beta-B, and Zr-beta-C were the key samples in this study, so their synthesis conditions are outlined in Table 1 for direct comparison. Zr-beta-A was prepared according to Wang et al.^36^ More specifically, 28.16 g of TEOS was mixed with 27.82 g of 40 wt % TEAOH (Sigma-Aldrich) and stirred for 2 h, subsequently dissolving 0.87 g of ZrOCl_2_·8H_2_O (98%, Sigma-Aldrich) in 3.86 g of water, which was added dropwise to the TEOS/TEAOH solution at room temperature. The resulting mixture was stirred for 3 h at room temperature. Then, 3.8 g of 40 wt % HF (VWR Chemicals) was added to the gel, followed by 0.28 g of beta seeds predispersed in 2.7 g of deionized water. The final synthesis gel had a molar composition of 100 SiO_2_/2 ZrO_2_/56 TEAOH/56 HF/1050 H_2_O. The gel hydrothermally crystallized at 140 °C under static conditions in a Teflon-lined steel autoclave for 20 days. After this period, the solid product was filtered, washed with 500 mL of deionized water, and dried at 60 °C. Calcination was performed in air at 580 °C (2 °C/min) for 10 h.

**Table 1:** Comparison of synthesis procedures for Zr-beta-A, Zr-beta-B, and Zr-beta-C.

	Zr-beta-A	Zr-beta-B	Zr-beta-C
synthesis gel composition (mol equivalent)^a^			
tetraethyl orthosilicate	100	100	100
tetraethylammonium hydroxide	56	53	56
water	1010	920	750
ZrOCl_2_ · 8 H_2_O	2		
ZrCl_4_		1	1
temperature during Zr source addition	22 °C	22 °C	0 °C
HF	56		56
beta seeds (% of total SiO_2_)	3.6%	8.6%	4.6%
HF		53	
hydrothermal synthesis conditions			
temperature	140 °C		
agitation	no		
synthesis time	20 days	18 days	13 days
calcination			
temperature	580 °C	550 °C	580 °C
temperature ramp	2 °C/min		
intermediate steps	none	none	150 °C (1h), 350 °C (1 h)
time at final temperature	10 h	6 h	10 h
atmosphere	air		

a The order of the compounds in the table follows their order of addition to the synthesis gel.

Zr-beta-B was prepared by mixing 35.17 g of TEOS with 37.59 g of 35 wt % TEAOH and stirring for 2 h, subsequently dissolving 0.36 g of ZrCl_4_ (99.9%, Sigma-Aldrich) in 1 g of water, which was added dropwise to the TEOS/TEAOH solution at room temperature. The resulting mixture was stirred for 3 h at 100 °C to evaporate ethanol. Weight loss was compensated for by adding distilled water. Then, 1 g of zeolite beta seeds was added at once, followed by 3.72 g of 48 wt % HF (VWR Chemicals). The final synthesis gel had a molar composition of 100 SiO_2_/1 ZrO_2_/53 TEAOH/53 HF/920 H_2_O. The gel hydrothermally crystallized at 140 °C under static conditions in a Teflon-lined steel autoclave for 18 days. After this period, the solid product was filtered, washed with 500 mL of deionized water, and dried at 60 °C. Calcination was performed in air at 550 °C (2 °C/min) for 6 h.

Zr-beta-C was prepared according to Wang et al.^37^ More specifically, 23.96 g of TEOS were mixed with 26.56 g of 35 wt % TEAOH and 15 g of distilled water and stirred for 2 h, subsequently dissolving 0.263 g of ZrCl_4_ in 2 mL absolute ethanol, which was added dropwise to the TEOS/TEAOH solution under stirring at 0 °C (ice bath). The resulting mixture was stirred for 48 h at room temperature to evaporate the ethanol. Weight loss was compensated for by adding distilled water. Then, 2.53 g of 50 wt % HF (Penta, the Czech Republic) was added dropwise, followed by 0.32 g of zeolite beta seeds. The final thick synthesis gel was homogenized with a PTFE spatula and had a molar composition of 100 SiO_2_/1 ZrO_2_/56 TEAOH/56 HF/750 H_2_O. The gel hydrothermally crystallized at 140 °C under static conditions in a Teflon-lined steel autoclave for 13 days. After this period, the solid product was filtered, washed with deionized water until reaching a neutral filtrate pH, and dried at 60 °C. Calcination was performed in air at 580 °C (2 °C/min) for 10 h with two intermediate isothermal steps at 150 and 350 °C for 1 h each.

Sn-beta-PS was prepared by degermanation and Sn insertion. The parent Ge-beta was prepared using a synthesis gel with a Si/Ge molar ratio of 6, according to Tosheva et al.^38^ The calcined Ge-beta was dispersed in 40 mL of a 0.1 M aqueous HCl solution (40 mL per 1 g of zeolite) and stirred at 85 °C for 16 h. The solid material was then filtered, washed with distilled water to pH = 7, and dried at 60 °C. In total, 863 mg of degermanated Ge-beta was activated at 450 °C for 90 min (heating rate 10 °C/min), cooled in a desiccator, and dispersed in a mixture of 0.63 mmol SnCl_4_ (1.0 M solution in heptane, Sigma-Aldrich) in 50 mL of anhydrous toluene (99.8%, Sigma-Aldrich). The mixture was stirred under a N_2_ atmosphere for 16 h at room temperature. Subsequently, the suspension was centrifuged, washed with anhydrous toluene twice, and dried in air at room temperature. The final product (Sn-beta-PS) was obtained after calcination at 550 °C (2 °C/min) for 8 h in air.

Na^+^ ion-exchanged Zr-beta-B (Na^+^ Zr-beta-B) was prepared as reported by Otomo et al.^39^ Briefly, calcined Zr-beta was dispersed in a 1.0 M NaNO_3_ solution (50 mL/g of zeolite). The resulting mixture was stirred at 80 °C for 12 h. Subsequently, the zeolite was separated by centrifugation, washed with distilled water, and redispersed in a 1.0 M NaNO_3_ solution. Ion exchange was repeated three times. After the third cycle, the washed sample was dried overnight at 100 °C and calcined at 500 °C for 5 h (2 °C/min).

Al-beta was purchased from Zeolyst International (Si/Al = 25, CP 814Q).

#### MFI Zeolites

Zr-MFI was synthesized as described by Zhao et al.^40^ In accordance with this protocol, 0.58 g of zirconium­(IV) isopropoxide (99.9%, Sigma-Aldrich) was dissolved in deionized water and added to 15.66 g of TEOS under stirring. Then, 25 mL of a 1 M tetrapropylammonium hydroxide solution (TPAOH, Merck) was added dropwise, and the mixture was stirred for 1 h. Lastly, 29 mL of distilled water was added to the mixture to obtain a clear homogeneous gel. Ethanol formed upon TEOS hydrolysis was evaporated at 60 °C for 2 h, and weight loss was compensated by adding distilled water. The final synthesis gel had a molar composition of 100 SiO_2_/2 ZrO_2_/33 TPAOH/4000 H_2_O. Hydrothermal crystallization was performed at 150 °C under agitation (60 rpm) in a Teflon-lined steel autoclave for 5 days. The solid product was filtered, washed with deionized water, and dried at 60 °C. This product was calcined in air at 550 °C (2 °C/min) for 7 h.

The pillared zeolite Zr-MFI-pill was prepared by silica metal-oxide pillaring^6^ of pure silica layered MFI zeolite, which was prepared following the procedure from reference^41^ but without using a Ti source. In a structure-directing-agent-containing form, the parent layered MFI was dispersed in a mixture of TEOS and zirconium­(IV) n-butoxide with a molar ratio of Si/Zr = 60 (this mixture was prepared by dropwise addition of zirconium­(IV) n-butoxide to TEOS and subsequent homogenization for 1 h using 10 g of the mixture per 1 g of zeolite). The pillar mixture with zeolite was stirred for 20 h at 65 °C in a closed flask. Subsequently, the mixture was centrifuged; the excess solution was poured out, and the sample was dried in a hood at room temperature for 48 h. The dry material was hydrolyzed in water with 5% ethanol (100 mL of solution per 1 g of solid material) for 24 h under continuous stirring. Lastly, the material was centrifuged, dried at room temperature, and calcined in an air flow at 550 °C (2 °C/min) for 8 h.

ZrO_2_ nanopowder was purchased from Sigma-Aldrich.

Al-MCM-41 was synthesized according to the procedure that was used to prepare sample A2, as reported in ref 42.^42^

### Characterization Methods

Textural properties were calculated from nitrogen adsorption–desorption isotherms acquired on a Micromeritics 3Flex Adsorption Analyzer at −196 °C. Prior to the analysis, the catalysts were outgassed under a turbo molecular pump vacuum (Micromeritics Smart Vac Prep instrument) at 250 °C for 8 h at a 1 °C/min heating rate. The Brunauer-Emmett-Teller (BET) area was calculated from data in the range *p*/*p*_0_ = 0.05–0.20, whereas micropore volume and external surface area were assessed using the t-plot method. Total adsorption capacity was determined from a single-point adsorbed volume at *p*/*p*_0_ = 0.95. The average pore diameter of Al-MCM-41 was assessed by nonlocal density functional theory (NLDFT) pore size analysis using model N_2_ on oxides at 77 K, included in the sorption instrument software.

The elemental composition of the catalysts was determined by inductively coupled plasma mass spectroscopy analysis on an Agilent 7900 ICP-MS and expressed as a Si/M (M = Zr, Sn, Al) molar ratio. Before each measurement, the samples were dissolved in a mixture of Aqua Regia and HF (4:1 vol/vol).

IR spectra were recorded at room temperature with a spectral resolution of 4 cm^–1^ on a Nicolet iS50 spectrometer equipped with a transmission MCT/B detector, and subtracting the background, collected with an empty evacuated cell. All samples were analyzed in the form of self-supporting wafers with a density ranging from 8 mg/cm^2^ to 12 mg/cm^2^. These samples were pretreated in an *in-situ* cell under vacuum (10^–3^ Pa) at 450 °C for 2 h prior to FTIR measurements. All spectra were normalized to the same sample density (10 mg/cm^2^) by multiplying them with a density factor calculated from the weight and area of each wafer. Consistent integrated areas of lattice T-O-T overtone and combination modes in the 1750–2100 cm^–1^ range confirmed the accuracy of the normalization.^43^ The adsorbed probe molecule band areas were calculated from subtracted, normalized spectra of each sample before and after the adsorption of each probe molecule.

Semiquantitative FTIR adsorption analysis of adsorbed *d*_3_-acetonitrile^21,44^ and acetone^23,45^ was performed to assess the type and strength of Lewis acid sites. For this purpose, the samples were saturated with an excess of *d*_3_-acetonitrile or acetone (except Zr-beta-C) at room temperature and 732 Pa. Subsequently, the cell was outgassed for 20 min at 25 °C for *d*_3_-acetonitrile and 50 °C for acetone to desorb excess physisorbed probe molecules. For Zr-beta-C, acetone was dosed in small portions until surface saturation to suppress the formation of diacetone alcohol, which had been observed when saturation was initially used with an excess of acetone.

Our powder X-ray diffraction (XRD) patterns were collected on a Bruker D8 Advance diffractometer equipped with a Linxeye XE-T detector, using Cu Kα radiation (λ = 0.15406 nm). The morphology and size of zeolite crystals (except Zr-MFI-pill and Al-MCM-41) were determined by scanning electron microscopy (SEM) under an FEI Quanta 200F microscope. The images were collected using an acceleration voltage of 20 kV. The Al-MCM-41 SEM image was collected under a JEOL, JSM-5500 LV microscope, also using an acceleration voltage of 20 kV, while the Zr-MFI-pill was analyzed under a Thermo Fisher Scientific Scios 2 DualBeam SEM microscope at an accelerating voltage of 2 kV.

### Catalytic Experiments

Catalytic reactions were performed at atmospheric pressure and 70 °C in a 25 mL three-neck round-bottom flask fitted with a reflux condenser, a magnetic stirrer, and a septum, placed in a Starfish workstation. The stirring speed was 450 rpm. Prior to the experiment, the catalysts were activated at 450 °C in air in a muffle oven for 6 h at 2 °C/min heating ramp. In a typical experiment, 100 mg of activated catalyst was added to 6 mL (78 mmol) of 2-propanol (99,7%, Lachner, Czech Republic). This mixture was heated to the reaction temperature; once the temperature stabilized, a mixture of 2.22 mmol of citronellal (96%, Alfa Aesar) and 1.29 mmol of mesitylene (99%, Sigma-Aldrich, internal calibration standard) was added to initiate the reaction. The reaction mixture was sampled every hour for 6 h. The samples were immediately cooled and centrifuged (4500 rpm for 5 min) to separate the catalyst. Subsequently, the samples were analyzed on an Agilent GC-7890 system equipped with VF-WAXms column (30 m × 0.25 mm × 1.0 μm) and a flame ionization detector, using nitrogen as a carrier gas. The products were identified by gas chromatography–mass spectrometry analysis on a Thermo Scientific Trace 1310 system equipped with a TG-5MS column (30 m × 0.25 mm × 0.25 μm) and by standard electron impact ionization.

## Results and Discussion

### Basic Physicochemical Properties of the Catalysts

The present study aimed at accurately correlating the catalytic activity of different Zr sites (i. e., “open” vs. “closed”) of beta zeolites with their selectivity in a system with competing reaction pathways. To this end, we (a) thoroughly characterized Zr Lewis acid sites in aluminum-free beta zeolites by FT-IR spectroscopy of adsorbed deuterated acetonitrile (*d*_3_-acetonitrile) and acetone and (b) assessed their catalytic performance in the citronellal reaction with 2-propanol in which MPV reduction competes with citronellal carbonyl-ene cyclization to isopulegol and acetalization. As outlined in Tables 1 and 2, we synthesized the following catalysts using various procedures: three catalysts with large pores, namely, (i) **Zr-beta-A** (rich in Zr “closed” sites), **Zr-beta-B** (rich in Zr “open” sites), and **Zr-beta-C** (containing both Zr “open” and “closed” sites), and the medium-pore zeolite reference catalyst (ii) **Zr-MFI** were all prepared by hydrothermal synthesis; the medium-pore zirconosilicate catalyst with enhanced active site accessibility (iii) **Zr-MFI-pill** was prepared by silica-zirconia pillaring^6,46^ of pure silica MFI nanosheets,^41^ thereby introducing Zr sites postsynthesis, primarily onto the external surface of the MFI nanosheets; and the Lewis acidic tin silicate beta zeolite catalyst (iv) **Sn-beta-PS** was prepared by degermanation and Sn insertion. Dealumination was replaced by degermination to prevent the leftover aluminum species from affecting the resulting catalytic properties. In addition, three other catalysts were used as references: **Al-MCM-41**, a high-silica mesoporous molecular sieve with amorphous walls containing a high concentration of silanol groups; aluminosilicate **Al-beta**, with both strong Brønsted and Lewis acid sites; and **ZrO**_**2**_, a Lewis acidic metal oxide.

**Table 2:** Bulk Si/M molar ratios and textural properties of the catalysts under study, including BET area (*S*_BET_), external surface area (*S*_ext_), micropore volume (*V*_micro_), and total adsorption capacity (*V*_total_).

sample		*S*_BET_	*S*_ext_	*V*_micro_	*V*_total_
sample	Si/M^a^	m^2^/g		cm^3^/g	
Zr-MFI-pill	22	601	190	0.10	0.47
Zr-MFI	30	325	153	0.15	0.30
Zr-beta-A	100	474	104	0.17	0.27
Zr-beta-B	130	567	111	0.20	0.32
Zr-beta-C	70	633	84	0.21	0.30
Sn-beta-PS	48	493	105	0.18	0.42
Al-beta	25	566	118	0.21	0.30
ZrO_2_	n.a.	31	30	0	0.13
Al-MCM-41	420	1035	96	0	0.84

a M = Zr, Sn, or Al; see sample notation; n.a. = not applicable.

Overall, powder XRD patterns (Figures S1 and S2, Supporting Information (SI)), elemental composition, and textural properties (Table 2) were consistent with data on these materials reported in the literature.^6,33,47^ Figure S3, SI, shows the N_2_ sorption isotherms of the catalysts and the corresponding BJH pore size distribution curves of Zr-MFI and Zr-MFI-pill. The Zr-MFI-pill catalyst is a composite of crystalline porous silica layers decorated with Zr^4+^ species and amorphous silica/zirconia pillars.^6^ The curves provide evidence of interlayer mesopores (pore diameter 3–4 nm) found in Zr-MFI-pill, but not in Zr-MFI.

Due to its lamellar structure, Zr-MFI-pill displayed broader diffraction lines than Zr-MFI. In the absence of a long-range order in the crystallographic *b*-direction, only *h*0*l* reflections were visible (Figure S1, SI).^48^ Al-MCM-41 showed the typical low-angle reflections of the hexagonal ordering of the pores^49^ (Figure S2, SI). No high-angle reflections were identified due to the amorphous walls.

SEM images of the catalysts (Figure S4, SI) showed morphologies typical of their materials. Zr-beta-A was characterized by 6–7 μm crystal agglomerates with visible terraces. Zr-beta-B, Sn-beta-PS, and Al-beta displayed 6–7 μm sponge-like crystal agglomerates. Zr-beta-C showed 6–7 μm crystal agglomerates of small (<200 nm) square crystals. Zr-MFI had regular, 200–300 nm, tablet-like crystals, whereas Zr-MFI-pill showed 1–2 μm layered agglomerates partly covered with amorphous silica from the pillaring treatment. Al-MCM-41 had 1–2 μm round particles.

In situ FT-IR spectroscopy enables us to observe (semi)­quantified surface −OH species and to identify and quantify molecules adsorbed on Lewis acid sites. Figure S5, SI, shows −OH stretching vibrations in FTIR spectra of the catalysts, indicating 4 types of −OH groups: isolated Si–OH groups in the external surface of the crystals (band at 3745 cm^–1^), isolated internal Si–OH groups located in the channels (band at 3735 cm^–1^), Si–OH-Al bridging hydroxyl groups of Brønsted acid sites (band at 3614 cm^–1^ in Al-beta spectra), and H-bonded Si–OH groups in silanol nests (broad band at ∼3500 cm^–1^ in Zr-MFI, Zr-MFI-pill spectra, and Al-MCM-41).^50^ The hydroxyl group content can be taken as a measure of the catalyst hydrophilicity. Therefore, Zr-MFI, Zr-MFI-pill, Al-MCM-41, and Sn-beta-PS are significantly more hydrophilic than Zr-beta-A, Zr-beta-B, and Zr-beta-C, which is in line with the preparation of these catalysts. Both silica pillaring and degermanation generate silanol defects, whereas direct hydrothermal synthesis of Zr-beta-A, Zr-beta-B, and Zr-beta-C yields fewer defective materials.

### Type and Strength of Zr Sites: FT-IR Study

The goal of our acidity study was to identify different Lewis acid sites in the Zr catalysts and to associate them with the observed catalytic properties. Differences between Zr-beta-A, Zr-beta-B, and Zr-beta-C samples are particularly relevant because they share other parameters, such as catalyst structure and channel size. In turn, aluminosilicate materials were not analyzed because they are well known for their Al Brønsted (Al-beta) and Lewis (Al-beta and Al-MCM-41) sites, which are significantly stronger than those resulting from Zr or Sn incorporation.^51,52^

*d*_3_-acetonitrile is a suitable probe molecule for differentiating Lewis acid sites of various types and strengths in zeolites.^21,43,44,53^ The position of the characteristic IR bands of LAS···*d*_3_-acetonitrile adducts formed during adsorption reflects the strength of Lewis acid sites.^43,53,54^ In Sn-beta, a band assigned to a ν­(C≡N) vibration is strongly blue-shifted, in comparison with its gas-phase position (2265 cm^–1^), due to the interaction with “open” (2315 cm^–1^) and “closed” (2304 cm^–1^) sites. In addition, a band at 2284 cm^–1^ was speculatively assigned to Sn double-defective sites,^43^ and *d*_3_-acetonitrile can also be hydrogen bonded to a silanol (2273 cm^–1^).^43,53,54^ Similar interactions of *d*_3_-acetonitrile were also observed on Zr, Hf, and Ti sites.^1^ In particular, interactions of *d*_3_-acetonitrile with Zr-beta give rise to a single band around 2306 cm^–1^. This band specifically probes the “open“ Zr sites, which was proved by pre-saturating Zr-beta with *d*_3_-acetonitrile and subsequently exposing Zr-beta to the CO probe.^21^ The CO band at 2176 cm^–1^, ascribed to “closed” sites, evolved in the spectra of both clean and *d*_3_-acetonitrile-presaturated Zr-beta, while the CO band at 2185 cm^–1^, ascribed to “open” sites, was strongly suppressed after *d*_3_-acetonitrile presaturation. Accordingly, *d*_3_-acetonitrile interacts with Zr “open” sites, but not with Zr “closed” sites for reasons that remain unclear. Identifying Zr “closed” sites required conducting an additional experiment using another probe molecule (see below).

The spectra of *d*_3_-acetonitrile adsorbed on Zr-beta-A, Zr-beta-B, and Zr-beta-C (Figure 1) contain bands arising from interactions with Zr “open” sites (2306 cm^–1^, Figure 1, green band), and silanols (2273 cm^–1^). Furthermore, the spectra contain a weak band at 2290 cm^–1^, which we speculatively ascribe to *d*_3_-acetonitrile hydrogen bonded to the Zr–OH group of the Zr “open” site because the ratio of its area to that of the 2306 cm^–1^ band was the same (0.19) across all Zr-beta samples. By contrast, the spectrum of the Zr-MFI contains an additional band at 2284 cm^–1^. Because *d*_3_-acetonitrile adsorption on Zr acid sites mirrors its adsorption on Sn acid sites, we preliminarily ascribed this additional band to double-defective Zr sites.

**Figure 1:**
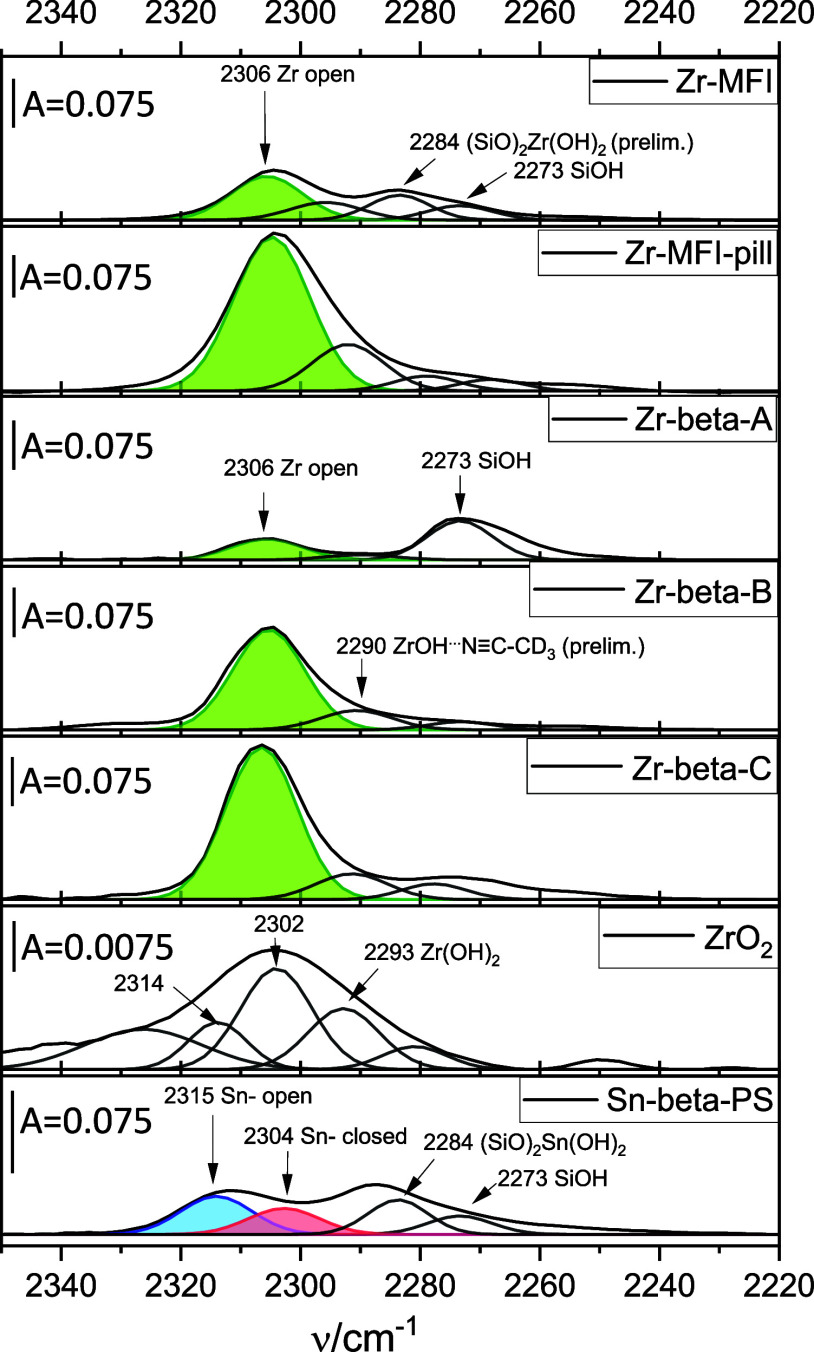
FTIR spectra in the stretching C≡N vibration region of zeolites interacting with *d*_3_-acetonitrile.

This ascription is arguable, since the position of this band is the same as that of the band speculatively ascribed to Sn double-defective sites. By contrast, the positions of the band assigned to “open” sites differ by 10 cm^–1^ between Sn-beta and Zr-beta (see above). However, the double-defective sites are weaker. Therefore, the actual difference in may be indistinguishable.

The spectra of Sn-beta-PS contain all the aforementioned characteristic bands,^53^ indicative of Sn “open” (Figure 1 blue band), Sn “closed” (Figure 1 red band), double-defective Sn sites, and silanol groups, respectively. The surface of bulk ZrO_2_ also contains a small amount of Lewis acid sites (note that the scale bar of the spectrum shown in Figure 1, ZrO_2_, is 10 times smaller). Differences in the corresponding band positions (ZrO_2_: 2314, 2302, and 2293 cm^–1^ vs. e.g., Zr-beta-A 2306, 2290, 2273 cm^–1^, Figure 1) demonstrate that the signals observed in the zeolites reflect their isolated Zr sites and not ZrO_2_ surfaces, thus confirming that Zr is incorporated into the zeolite framework.

The SiOH···*d*_3_-acetonitrile band at 2273 cm^–1^ was stronger in Zr-beta-A than in Zr-beta-B and Zr-beta-C, even though the spectra of these zeolites were similar in the region of −OH stretching vibrations (Figure S5, SI). Nevertheless, the intensity and shape of the Si–OH bands depends on factors such as hydrogen bonding, silanol clustering, and local acidity.^55^ For this reason, materials with different Si–OH populations may display nearly indistinguishable OH stretching regions, as found in these materials.

Although the bulk Zr content of the Zr-beta-A (Si/Zr = 100) and Zr-beta-B (Si/Zr = 130) samples was similar, the area of the 2306 cm^–1^ band—associated with Zr “open” sites—differed significantly between them. After normalizing the area of the 2306 cm^–1^ band to the bulk molar Zr content (by Zr/Si molar ratio ×100, Table 2), we found the following order: Zr-beta-B (3.08)>Zr-beta-C (2.52)>Zr-beta-A (0.49). Accordingly, the share of “open” sites on all Zr atoms is the highest in Zr-beta-B and the lowest in Zr-beta-A. The spectra of Zr-MFI-pill (Zr normalized 2306 cm^–1^ band area of 0.85) and Zr-MFI (0.42) show that their share of “open” sites is similar to that of Zr-beta-A (likely a minority of all Zr species). But this low share of “open” sites may be caused by Zr trapping inside silica pillars during the silica-zirconia pillaring, especially in Zr-MFI-pill. In any event, the key question as to whether the remaining share of Zr formed the “closed” sites or any other Zr species was still not answered.

To answer this question, we could have used CO adsorption to distinguish “open” and “closed” Zr sites.^21^ Instead, we selected acetone as the probe molecule, because its adsorption more closely mimics that of citronellal, our target reagent. Acetone provides a polarized C=O functional group and interacts with acid sites in a manner more representative of larger oxygen-containing reactants. In contrast, CO offers only a simple σ-donor-π-acceptor interaction and is unable to capture steric effects relevant to oxygenated substrates. Acetone molecules interact with zeolites mainly in 3 ways: (i) weak hydrogen bonding on Si–OH groups, (ii) coordination through the lone electron pair of the carbonyl group on Lewis acid sites, and (iii) strong hydrogen bonding to Brønsted sites when present (not applicable to Lewis acid zeolites).^23,45,56–59^ In addition, adsorbed acetone undergoes aldol condensation, yielding diacetone alcohol, which can be easily dehydrated to mesityl oxide (Figure 2, bottom).

**Figure 2:**
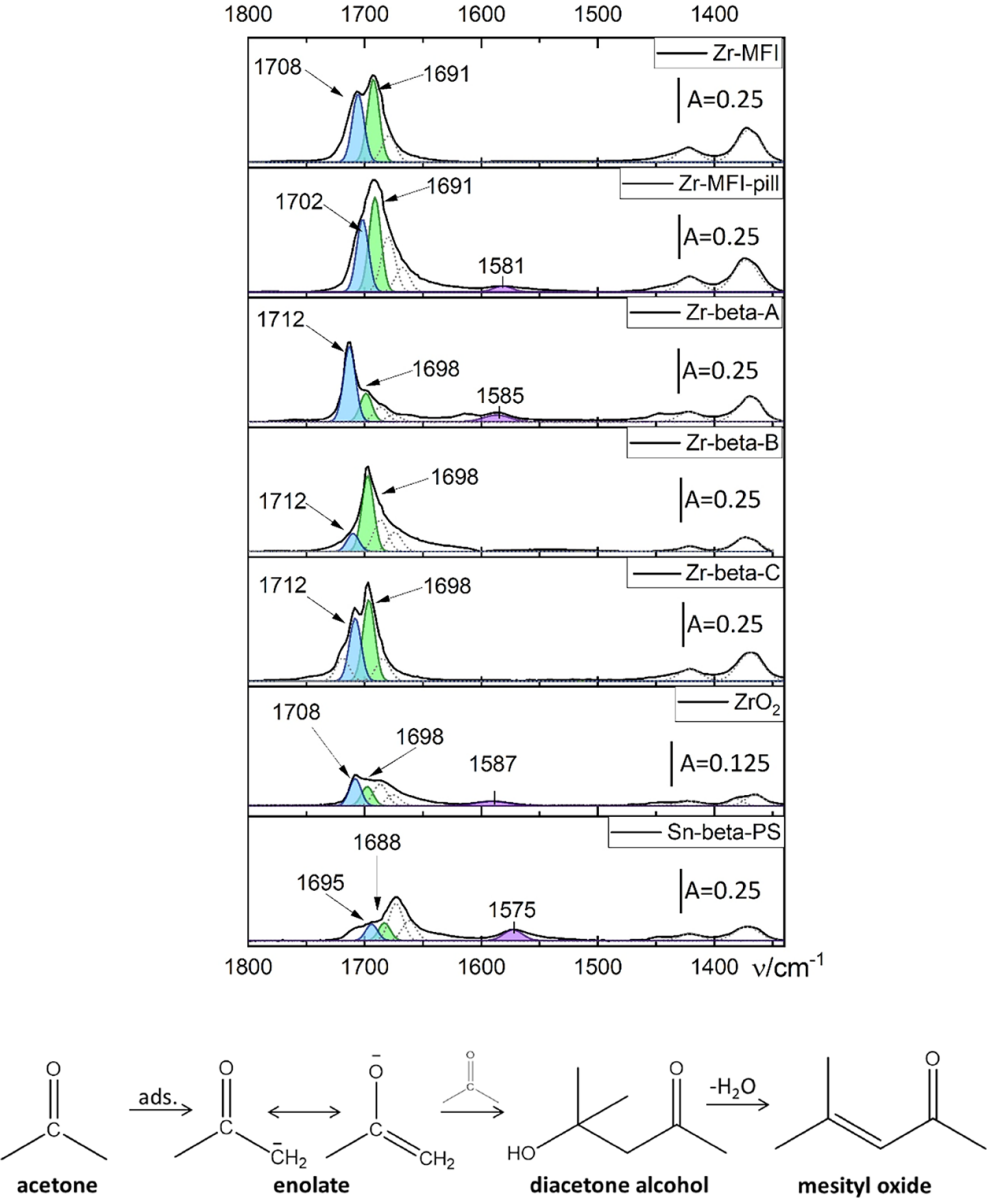
FTIR spectra in the stretching C=O vibration region of zeolites interacting with acetone (top); scheme of aldol addition to acetone and diacetone alcohol dehydration to mesityl oxide^59^ (bottom).

Aldol condensation occurs particularly over Lewis basic sites near a Lewis acid site, which is the initially adsorption site (framework oxygen can act as the basic site), or at high temperatures (>200 °C).^60^ Mesityl oxide can further react with another acetone molecule, forming phorone, which can be further transformed to isophorone and finally aromatics; however, these condensations were observed only at high temperatures, so they did not occur under our experimental conditions.^23,61^ To remove acetone molecules physisorbed on the catalyst surface and to minimize the formation of diacetone alcohol and mesityl oxide adsorbed species (evidenced by bands in the 1650–1500 cm^–1^ range^60,62^ ), the samples were outgassed at 50 °C for 20 min after acetone adsorption.

Figure 2 shows spectra of acetone adsorbed on the catalysts, displaying red-shifted (conversely, *d*_3_-acetonitrile adsorbed on Lewis acid sites exhibits a blueshift) bands of acetone molecules interacting with acid sites, in relation to the gas-phase vibration position (1731 cm^–1^).^63^ This redshift is a general feature of carbonyl molecules, except for CO.^13,22^ The overlapping features found between 1750 and 1600 cm^–1^ were characteristic of the stretching vibration of the carbonyl group and indicated that more than one type of adducts were chemisorbed on the catalyst surface. The symmetric and asymmetric bending vibrational modes of the methyl groups of adsorbed acetone were located at 1375 and 1420 cm^–1^, respectively. The band at ∼1580 cm^–1^ was attributed to the ν­(C=C) of mesityl oxide, and two other bands at 1680 and 1672 cm^–1^ were assigned to the stretching vibration of the carbonyl group of diacetone alcohol and mesityl oxide, respectively.^22,23,59^ These products result from acetone condensation on the catalyst surface. The bands at the highest wavenumbers (1712–1690 cm^–1^) were assigned to the ν­(C=O) vibration of acetone molecules interacting with Lewis acid sites of the zeolites.^59,61,62^

By observing the evolution of spectra upon gradual acetone adsorption (measured on Zr-beta-C, Figure 3) dose by dose, we ascribed the bands to Lewis acid sites of different strength. The band at 1698 cm^–1^ (at 1691 cm^–1^ for Zr-MFI), which evolved first, was attributed to acetone interacting with stronger Lewis sites.^22^ The second band to evolve, located between 1709 and 1712 cm^–1^, was assigned to a second, weaker type of acid sites. The third band, at 1719 cm^–1^, was ascribed to acetone, either physisorbed (note that its position matches the position of acetone ν­(C=O) in liquid phase)^59^ or H-bonded to silanols. After evacuation, the band at 1719 cm^–1^ vanished. Acetone adsorption and subsequent evacuation of the Al-MCM-41 confirmed that none of the bands between 1712 and 1691 cm^–1^ resulted from adsorption on silanol groups as no acetone band was observed after Al-MCM-41 evacuation at 50 °C (Figure S6, SI).

**Figure 3:**
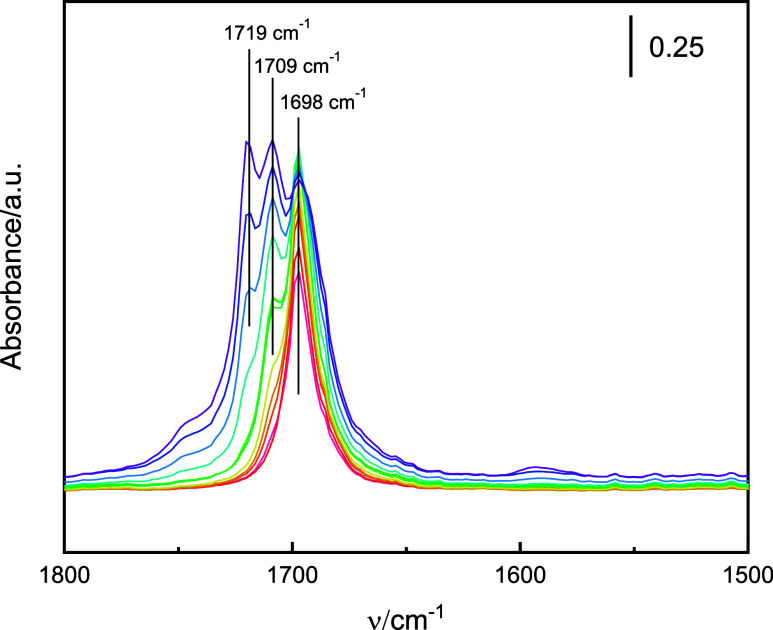
Gradual adsorption of acetone on Zr-beta-C at room temperature shows the evolution of the bands characteristic of acetone adsorption on sites with different strengths; the strongest sites (1698 cm^–1^) are occupied first. Spectra after every 2nd dose are presented following the visible color spectrum: the first dose is shown in red, and the last dose in violet.

The 20–40 cm^–1^ redshifts of acetone ν­(C=O) vibrations correspond to similar adducts of cyclohexanone on Sn (shift of 48 cm^–1^) and Ti (32 cm^–1^) Lewis sites.^13^ Considering that the strongest sites are occupied first and that Zr “open” sites are stronger than “closed” sites,^21,43,51^ we assigned the band at 1698/1691 cm^–1^ to acetone molecules coordinated to “open” sites, and the band at 1712–1708 cm^–1^ to the C=O vibration of acetone molecules on Zr “closed” sites. The corresponding values of the bands observed at Sn-beta-PS are 1688 cm^–1^ for Sn “open sites” and 1695 cm^–1^ for the Sn “closed sites”. The 1698 cm^–1^ band area also correlates with the area of the *d*_3_-acetonitrile band characteristic of Zr “open” sites (2306 cm^–1^, Figure S7, SI).

The bands below ∼1690 and 1680 cm^–1^ for Zr and Sn zeolites were not observed by dose adsorption after the primary dose. These bands likely correspond to products of the acetone surface reactions. Once again, the surface of bulk ZrO_2_ also showed a low concentration of Lewis acid sites.

### The Type of Active Sites Explains the Catalytic Performance

The Lewis acid-catalyzed reaction of citronellal with/in 2-propanol in liquid phase at 70 °C can follow three main reaction pathways: (i) MPV reduction, yielding citronellol and acetone; (ii) intramolecular carbonyl-ene cyclization, yielding a pool of isopulegol isomers; and (iii) acetalization, yielding citronellal diisopropylacetal (Scheme 2). In addition to these products, several minor products were identified as citronellal isomerization products (hereafter denoted as “others”). Table 3 lists single-point conversion and yield data on all catalysts after reaction for 6 h.

**Table 3:** Citronellal (2.2 mmol) reaction with/in 2-propanol (78 mmol) over 100 mg of catalyst at 70 °C; citronellal conversion and product yields are given after 6 h.

		yield (%)			
catalyst	conversion (%)	isopulegol	citronellol	acetal	others
Zr-MFI-pill	96	73	1.8	12	9.2^e^
Zr-MFI	18	8.2	1.6	7.8	0
Zr-beta-A	83^a^	13	66	0	4
Zr-beta-B	100^b^	76	17	3	2.5
Zr-beta-C	100^c^	48	49	1.7	1.8
Sn-beta-PS	98	82	1.6	8	6.4
Al-beta	100^b^	80	0	8	10
ZrO_2_	0	0	0	0	0
Al-MCM-41	77	18	0	59	0
Na^+^ Zr-beta-B^d^	78	13	55	3.9	8.6
no catalyst	0	0	0	0	0

a data given after 5 h of reaction

b 100% conversion reached within 1 h, after which time the product distribution did not change.

c 100% conversion reached within 2 h, after which time the product distribution did not change.

d Na^+^ ion-exchanged Zr-beta-B

e mostly citronellal isopropyl hemiacetal

As expected, the channel size and thus active site accessibility determined the conversion. The large-pore zeolites, Zr-beta-A, Zr-beta-B, and Zr-beta-C, reached 83, 100, and 100% conversion, respectively. A similar conversion level was observed when using the reference zeolite Sn-beta-PS (98%). The reference zeolite Al-beta afforded a total conversion in 1 h. With medium pores, Zr-MFI provided only 18% conversion despite containing more Zr than Zr-beta-A, Zr-beta-B, and Zr-beta-C (Zr-MFI Si/Zr = 30 vs. 100, 130, 70, respectively). With similar Zr content (Si/Zr = 40) but improved active site accessibility, the lamellar analogue of Zr-MFI, Zr-MFI-pill, provided 96% conversion. Bulk ZrO_2_ did not catalyze citronellal transformation.

The hydrothermally synthesized Zr-beta zeolites strongly differed in product yields (Zr-beta-A: 13% isopulegol yield, 66% citronellol yield; Zr-beta-B: 76% isopulegol yield, 17% citronellol yield; Zr-beta-C: 48% isopulegol yield, 49% citronellol yield, after 6 h), although their structure, overall Zr content, and textural properties were the same or similar (Table 2). Figure 4 shows the variation of the product yield as a function of time over the Zr-beta-A, Zr-beta-B, and Zr-beta-C catalysts. Based on the shape of the yield curves, isopulegol, citronellol, and citronellal diisopropylacetal (hereafter acetal) are formed in parallel reactions, and once citronellal is consumed, the composition of the reaction system does not change, so the products do not further transform under these reaction conditions. For instance, Zr-beta-A isopulegol selectivity is 15%, whether at 35% (sample taken at 1h) or 100% conversion (sample taken at 24 h). In other words, the reaction selectivity is conversion independent (cf. also isopulegol selectivity curves in Figure S8, SI). Accordingly, either at least two types of active sites catalyze the carbonyl-ene cyclization and MPV reactions to different extents or each one of the reactions is catalyzed by one type of active sites. Correlating the catalytic activity with the IR acidity analysis helped us to find the most appropriate explanation. Linear selectivity curves (Figure S8, SI) also ruled out changes in active sites during the catalytic run.

**Figure 4:**
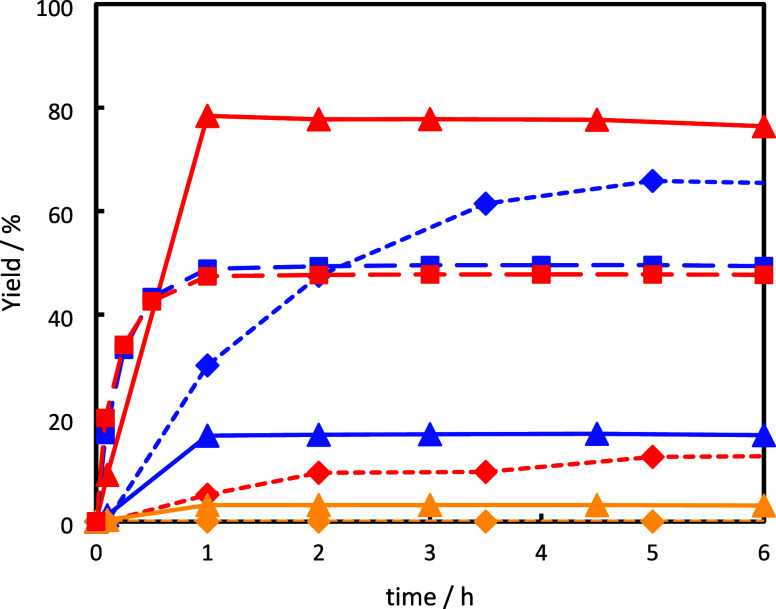
Variation of isopulegol (red), citronellol (blue), and citronellal diisopropylacetal (yellow) yield over Zr-beta-A (◊, dashed lines), Zr-beta-B (Δ, straight lines), and Zr-beta-C (□, dashed lines) as a function of time; the curve of citronellal diisopropylacetal produced over Zr-beta-C is omitted for clarity.

Zr-beta-A contains a significantly higher concentration of “closed” sites than Zr-beta-B (1712 cm^–1^ band area of Zr-beta-A 8.78 arbitrary units (a.u.) vs. of Zr-beta-B 2.10 au, Figure 2) and a lower concentration of “open” sites. Conversely, Zr-beta-B is rich in “open” sites (Figures 1,2), and Zr-beta-C stands in between. To some extent, the catalysts differ in Si/Zr ratio, morphology (which affects overall conversion, cf. Table 2 and Figure S4, SI), and Si–OH coverage (which mainly affects the acetalization pathway; see below). But this difference in reaction selectivity (e.g., selectivity to isopulegols 15% Zr-beta-A vs. 78% Zr-beta-B, at 100% conversion) may derive from the difference in content between the two types of Lewis sites. The weaker, “closed” sites catalyze the MPV reaction, yielding citronellol, while the stronger, “open” sites catalyze the intramolecular carbonyl-ene cyclization, yielding isopulegol isomers, so the relative share of closed vs. open sites determines the reaction selectivity (Figure 5). Our analysis focuses on the selectivity of the MPV and carbonyl-ene pathways evaluated at identical conversions. The comparison is justified under these conditions.

**Figure 5:**
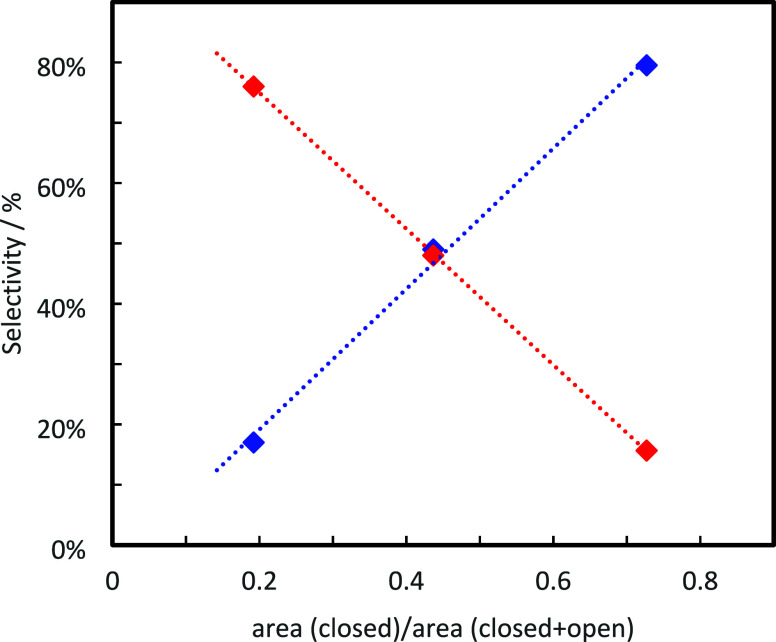
Variation of isopulegol (red) and citronellol (blue) selectivity (at 100% conversion) as a function of the relative area of acetone bands ascribed to closed sites (1712 cm^–1^) and to the sum of closed and open sites (1698 cm^–1^).

Because each type of site (“open”/”closed”) is responsible for a specific reaction, we were able to selectively deactivate one type of active sites. Zr and Sn “open” sites are sometimes denoted Brønsted sites, although they are too weak to protonate, e.g., pyridine. Nevertheless, these “open” sites are ion-exchangeable.^39,64^ Therefore, we deactivated the Zr “open” sites by ion exchange with Na^+^ ions.

Following a procedure reported by Otomo et al.,^39^ Zr-beta-B ion-exchanged with NaNO_3_ (Na/Zr molar ratio = 0.59 after the ion exchange) provided 78% conversion after 6 h, with yields of 55% citronellal and 13% isopulegol (Table 3). When we calculated the initial turnoverfrequency of citronellol and isopulegol formation from the first data point (Figure S9, SI), the TOF of isopulegol formation dropped from 157 to 10 h^–1^ after ion exchange. In contrast, the TOF of citronellol formation (MPV reduction) increased from 21 to 65 h^–1^ after ion exchange. This difference suggests that at least some of the sites that originally catalyzed isopulegol formation catalyze MPV reduction upon ion exchange. And while the increase in MPV reduction TOF is not proportional to the decrease in carbonyl-ene reaction TOF, this shift highlights a potential direction for engineering Zr-beta catalytic properties after synthesis. A control experiment with only NaNO_3_ (without zeolite) resulted in zero conversion. This selectivity switch upon ion exchange of “open” Zr sites supports our assertion that the Zr-site Lewis acidity determines terpenoid reduction selectivity.

Our assertion is also in line with the properties of reference catalysts. For example, glucose isomerization, which is an intramolecular MPV reaction, is ascribed to Sn “open” sites,^43,65^ but Sn-beta “open” and Zr-beta “open” Lewis sites show similar acetone interaction energies (−53 kJ/mol).^51^ As such, Sn “open” sites should catalyze carbonyl-ene cyclization as effectively as Zr “open” sites in the citronellal transformation, as observed in this study.

With Lewis acid sites stronger than Zr sites, Al-beta provided no citronellol. Once again, this finding demonstrates that strong “open” sites catalyze carbonyl-ene cyclization, yielding isopulegol. But when the substrate structure (e.g., *tert*-butylcyclohexanone) allows no reaction other than MPV, even Al-beta provides the MPV reaction product (*tert*-butylcyclohexanol).^34^ Thus, similarly to Sn “open” sites, Zr “open” sites can catalyze both carbonyl-ene cyclization and the MPV reaction but favor the former over the latter.

In contrast to Zr-beta, the Zr-MFI and Zr-MFI-pill catalysts provided practically no citronellol. Since the spectra of adsorbed acetone (Figure 2) were similar to that of Zr-beta-C, the high isopulegol/citronellol yield ratio (Table 3) may be attributed to the lack of space in channels to accommodate the bulky transition state of the MPV reaction. This transition state involves simultaneous coordination of both reactants to the acid site.^66^ Therefore, the reaction selectivity is defined by the size of the transition state rather than by the prevailing type of acid sites.

### Acetal Formation Is Facilitated by Silanols

Zr-MFI-pill provided a considerable acetal yield (12%). Acetal is the bulkiest possible product, so its formation may not occur in zeolite micropores but may be associated with amorphous silica pillars and more broadly with silanol nests because Sn-beta-PS reached 8% yield of acetal (note the correlation with the aforementioned observation of the IR band at 3500 cm^–1^). Conversely, almost no acetal was formed over hydrothermally synthesized Zr-beta catalysts and no acetal was formed in a blank experiment. Acetalization is a H^+^-catalyzed reversible reaction, yielding water as a side product. In this study, we detected stable acetal, even at total citronellal conversion (Table 3). Zeolite silanols catalyze acetalization;^67^ however, beyond providing Brønsted acid sites, our catalysts must also act as water scavengers because acetal does not fully decompose even when total citronellal conversion is reached over Zr-MFI-pill and other catalysts (cf. Table 3). To test our hypothesis that silanols play a key role as water scavengers, we used an Al-MCM-41 (Si/Al = 420) catalyst.

Al-MCM-41 is a mesoporous molecular sieve with amorphous silica walls, a high concentration of silanol groups (Figure S5, SI), and an average pore size of 3.8 nm. Thus, its channels should not restrict any of the reaction pathways. In this study, Al-MCM-41 provided 77% conversion and 60% acetal yield (together with 18% isopulegol yield formed over trace amounts of Al sites) in 6 h. These results corroborate the findings of Koehle and Lobo,^14^ according to whom the reaction rate of the furfural diisopropylacetal side product formation does not depend on the heteroelement (Sn, Zr, or Hf) of zeolite beta catalysts in the MPV reduction of furfural.

We also observed that the Al-beta reference provided a lower acetal yield (8%) than Al-MCM-41 (59%) after 6 h (Table 3) despite containing many more Al sites than Al-MCM-41 and that Al-free Zr-MFI-pill also provided a 12% acetal yield. Based on these results, acetalization cannot be ruled out in the absence of strong Al acid sites, so silanols catalyze acetalization, and the resulting water remains trapped on the catalyst surface. The more silanols are available, the more water can be trapped, increasing the acetal yield.

## Conclusions

Weak Zr-beta “closed” sites catalyze the MPV reduction of citronellal, whereas strong Zr-beta “open” sites catalyze intramolecular carbonyl-ene cyclization. The silanol groups of this catalyst promote acetalization regardless of the substituting heteroelement. Bulk ZrO_2_ is inactive under these reaction conditions. These findings are consistent with the catalytic results of reference zeolites, namely, Sn-beta, Al-beta, and Al-MCM-41. Ion exchange of Zr-beta rich in “open” sites with Na^+^ cations deactivates “open” sites, thus switching the selectivity to citronellol as the main product. In contrast, Zr-MFI and Zr-MFI-pill do not yield citronellol because their medium-sized pores prevent the formation of citronellol, given the lack of space to accommodate the bulky bimolecular transition state of the MPV reaction. So, as long as the zeolite channels are sufficiently wide (zeolite beta), the type of Zr Lewis site determines the product distribution. Ascribing reaction pathways to specific types of acid sites furthers our understanding of zirconosilicate zeolite catalytic properties and may facilitate the design of specific catalysts, even for systems with competing reactions, by acquiring quantitative data using this experimental paradigm.

## Supplementary Material



## Data Availability

The presented data are available to download from the Zenodo re­pository at https://doi.org/10.5281/zenodo.18471633.

## Notes

Additional catalyst characterization data; including XRD patterns; N_2_ physisorption isotherms; additional IR spectra; and catalytic data (PDF)
