# Supplementary material for: Zr-Site Lewis Acidity Determines Terpenoid Reduction Selectivity
Source: ACS Catal. 2026 Feb 5;16(4):3307–18. doi: 10.1021/acscatal.5c07220 (PMC12930520; doi:10.1021/acscatal.5c07220)
Supplement: Supplementary file 1 [file cs5c07220_si_001.pdf]

Supporting information

Zr-site Lewis acidity determines terpenoid reduction selectivity

Kinga Gołąbek, Svetlana Kurucová, Juan Francisco Miñambres, Klára Veselá, Talat Zakeri and Jan Přech*

Department of Physical and Macromolecular Chemistry, Faculty of Science, Charles University,

Albertov 6, 128 43, Praha, Czech Republic

*Corresponding author, e-mail: jan.prech@natur.cuni.cz, phone: +420 221 95 1322

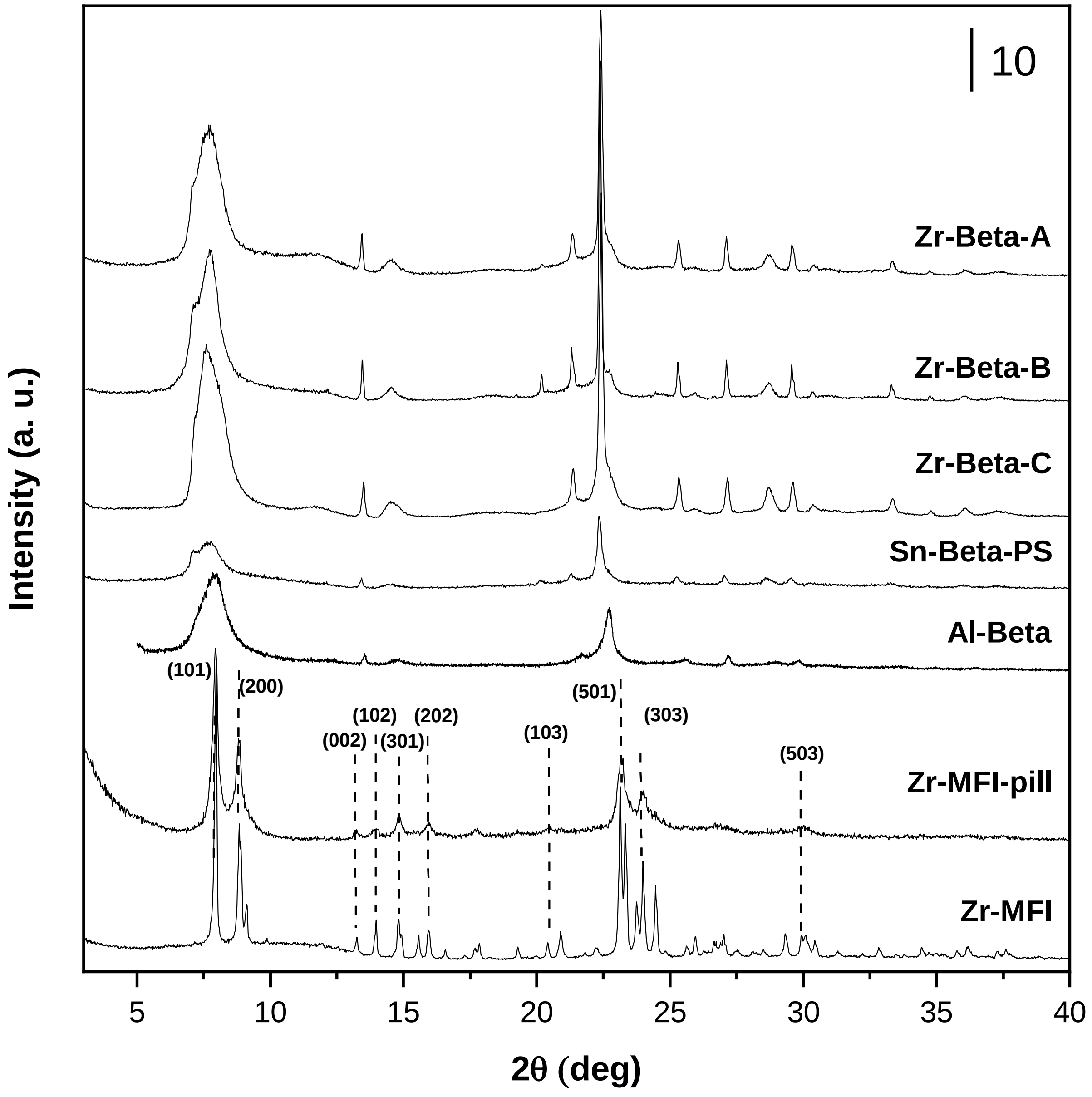

Figure S 1: XRD patterns of the catalysts under study; (h0l) lines of the MFI patters are marked for clarity.

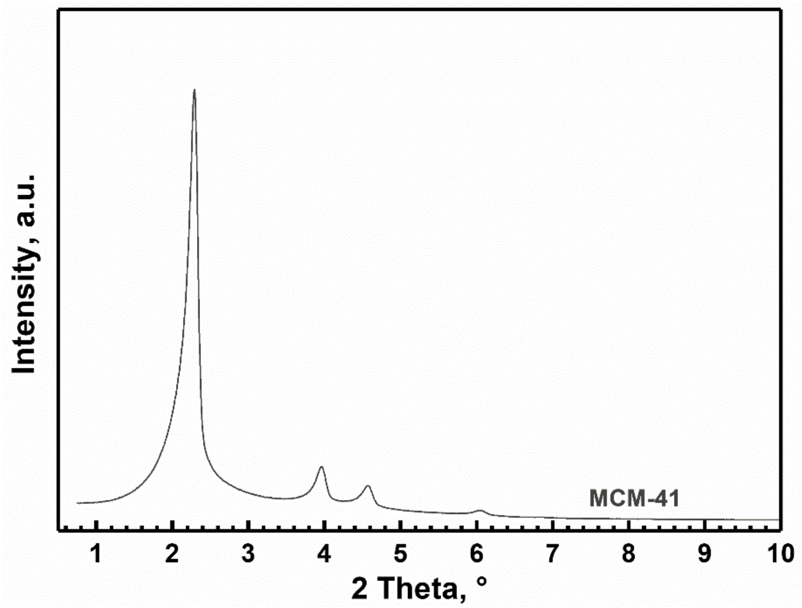

Figure S 2: XRD pattern of Al-MCM-41

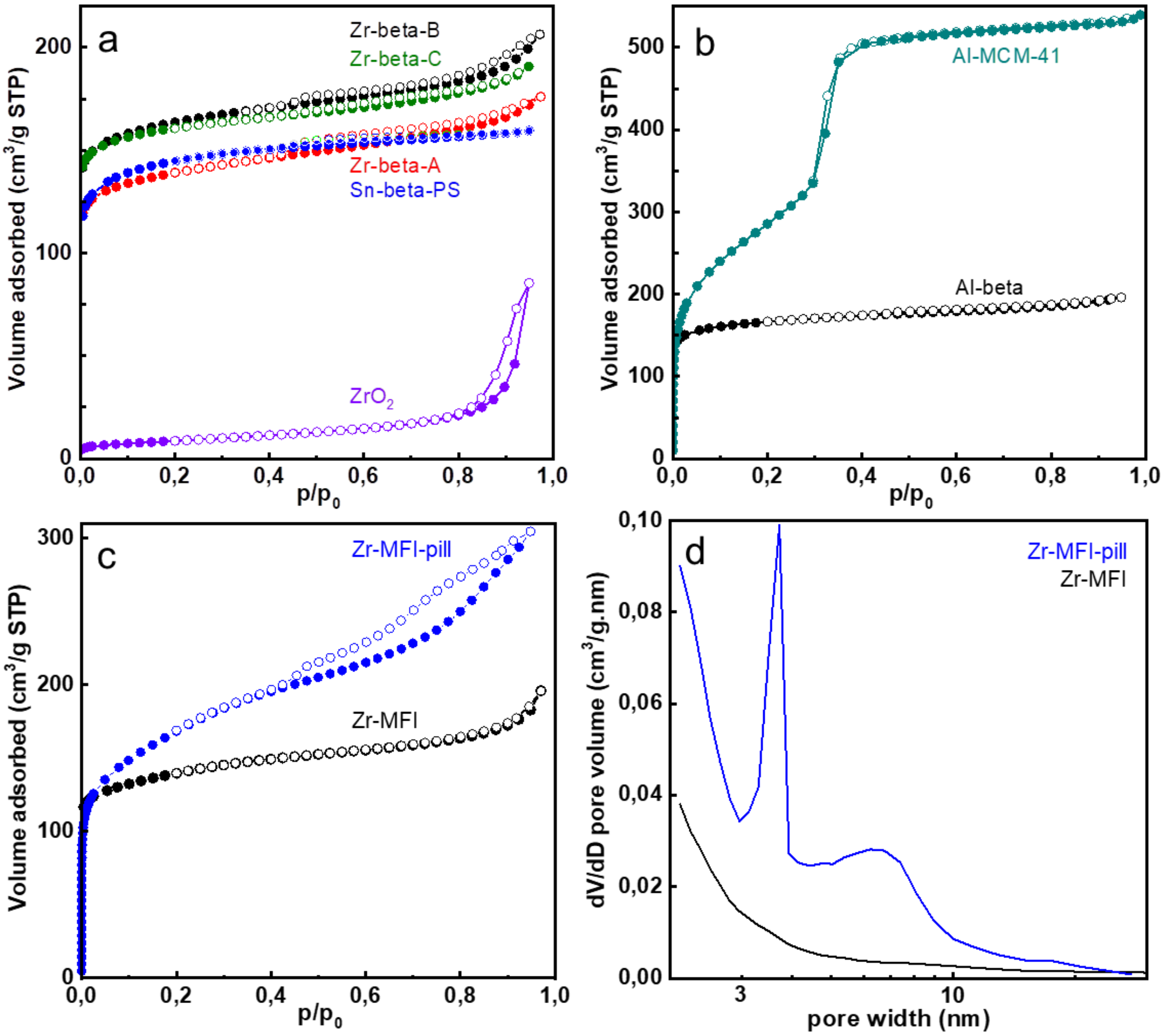

Figure S 3: N_2 sorption isotherms of the catalysts (a-c) and BJH pore size distribution curves of Zr-MFI and Zr-MFI-pill (d); empty circles denote desorption.

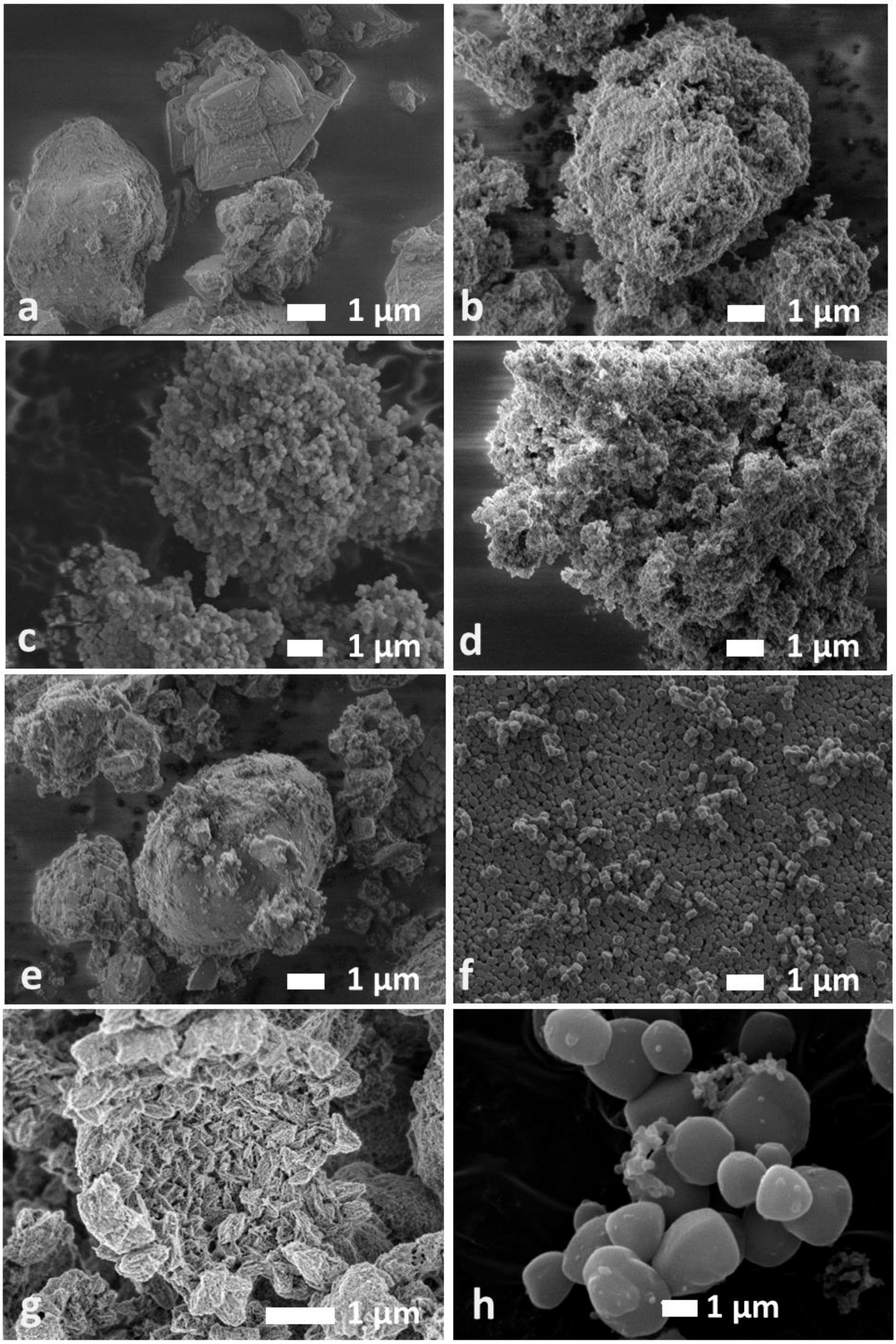

Figure S 4: SEM images of the Zr-Beta-A (a), Zr-Beta-B (b), Zr-Beta-C (c), Al-Beta (d), Sn-beta-PS (e), Zr-MFI (f), Zr-MFI-pill (g) and Al-MCM-41 (h).

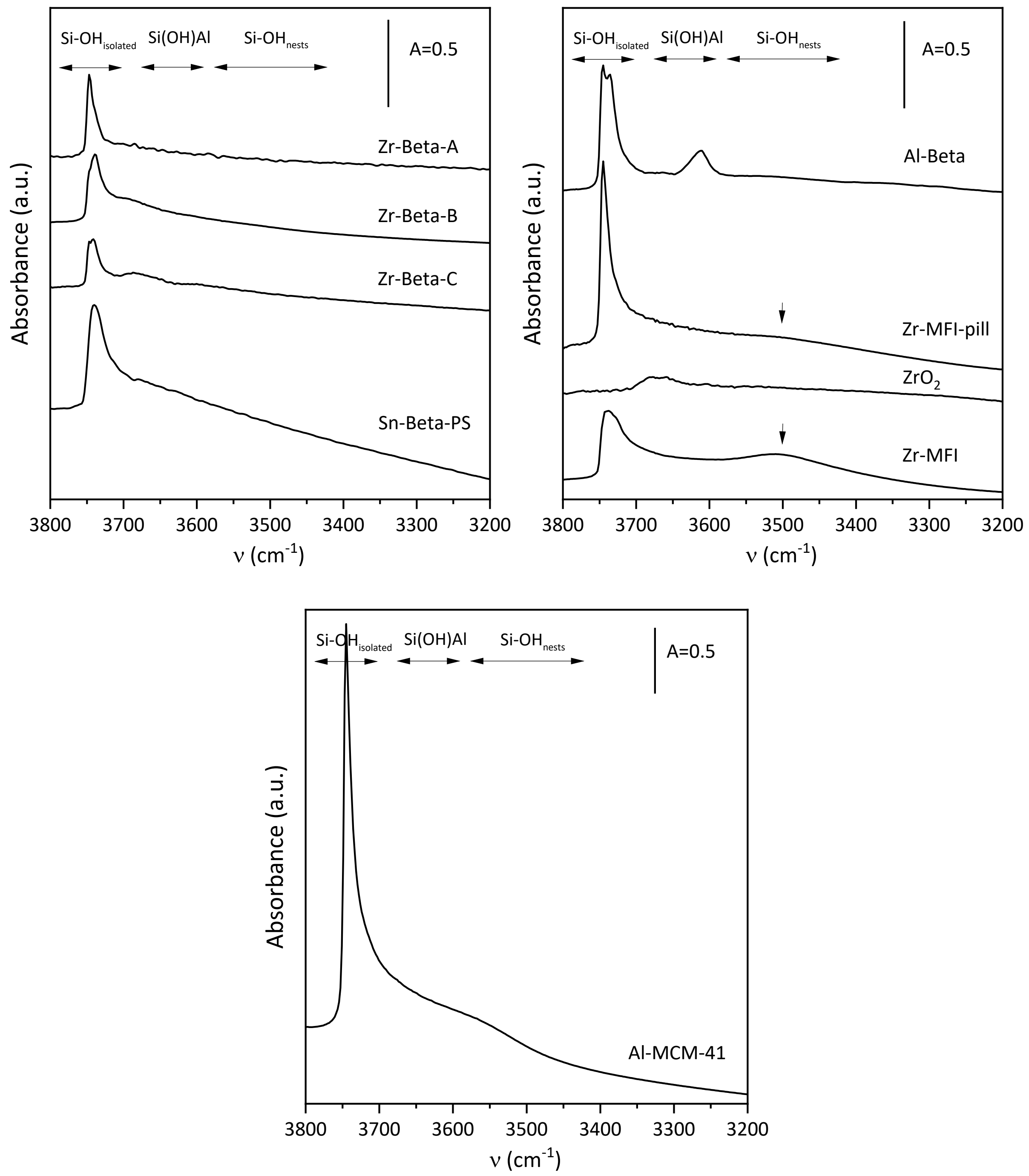

Figure S 5: FTIR spectra of catalysts in the region of -OH stretching vibrations; arrows highlight a broad band at 3500 cm^{-1} characteristic of internal silanol defects (silanol nests).

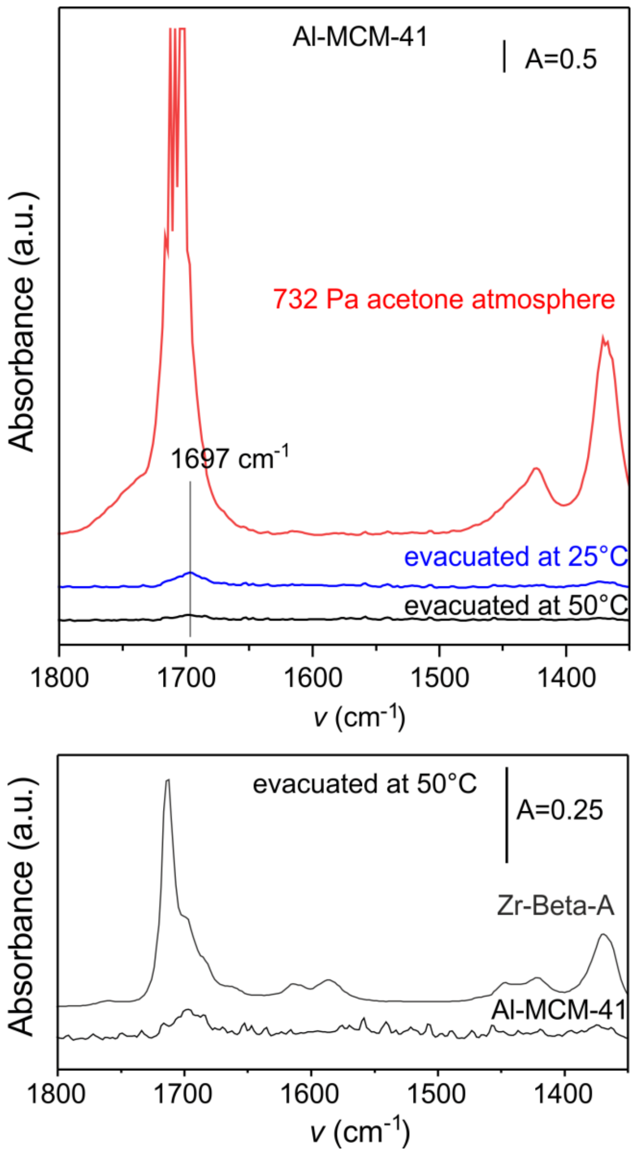

Figure S 6: Acetone adsorption and desorption IR spectra of Al-MCM-41 (top) and comparison of the Al-MCM-41 spectrum after desorption at 50°C with that of Zr-beta-A (bottom) show that no acetone remains adsorbed on Al-MCM-41 after desorption at 50°C. Thus, none of the bands ascribed to acetone coordinated to Zr-sites result from adsorption on silanol groups.

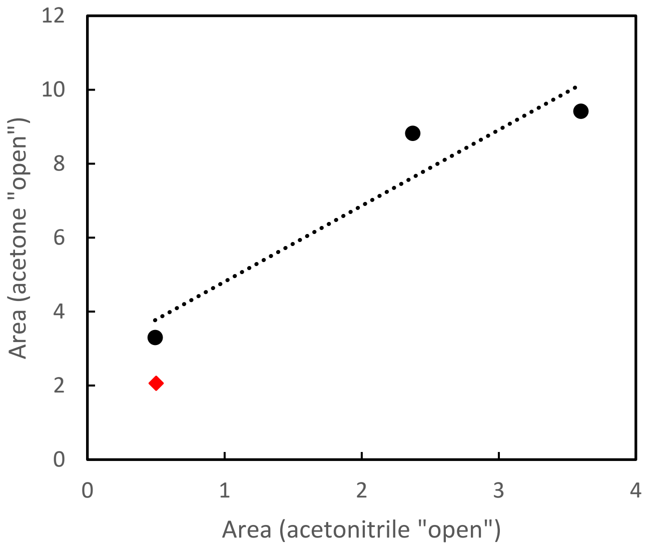

Figure S 7: Correlation of the band area of "open" Zr sites probed by acetonitrile (2305 cm^{-1}) and acetone (1698 cm^{-1}) in Zr-beta-A, B, and C samples. For comparison, red squares show the relative band area of "open" Sn sites (2316 and 1688 cm^{-1}, respectively) in the reference sample Sn-beta-PS.

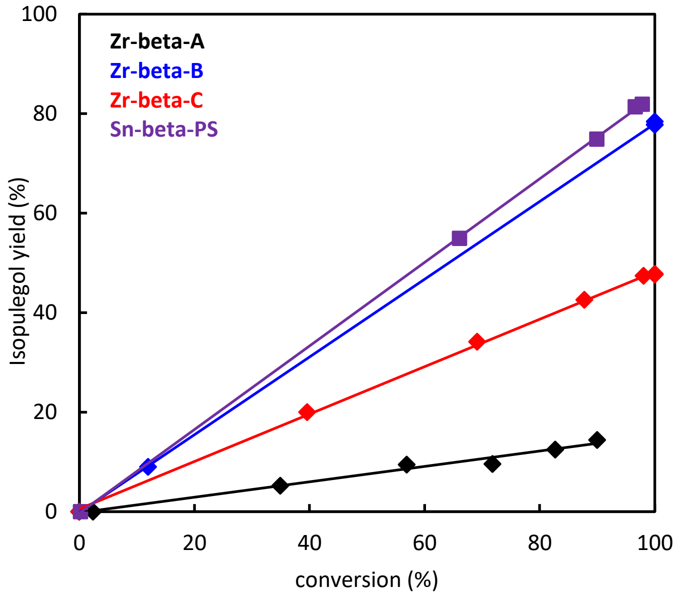

Figure S 8: Isopulegol selectivity curves of Zr-beta-A, B, and C and Sn-beta-PS catalysts showing no changes in selectivity during the catalytic run

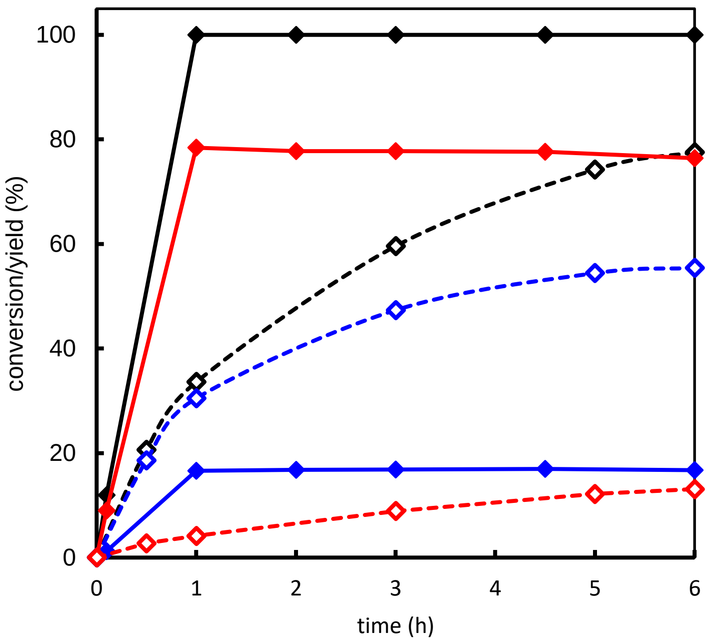

Figure S 9: Variation of citronellal conversion (black) and isopulegol (red) and citronellol (blue) yield over Zr-beta-B (solid points, straight lines) and Na+ Zr-beta-B (empty points, dashed lines) as a function of time
